# Probing subicular inputs to the medial prefrontal cortex

**DOI:** 10.1016/j.isci.2021.102856

**Published:** 2021-07-10

**Authors:** Sanne Beerens, Rozan Vroman, Jack F. Webster, Christian Wozny

**Affiliations:** 1 Strathclyde Institute for Pharmacy and Biomedical Sciences, University of Strathclyde, Glasgow, UK; 2 MSH Medical School Hamburg, Faculty of Medicine, Medical University, Hamburg, Germany

**Keywords:** Anatomy, Animal physiology, Neuroscience

## Abstract

The hippocampal formation is anatomically and functionally divided into a dorsal and a ventral part, being involved in processing cognitive tasks and emotional stimuli, respectively. The ventral subiculum as part of the hippocampal formation projects to the medial prefrontal cortex (mPFC), but only very little is known about connections arising from the dorsal SUB (dSUB). Here, we investigate the dSUB to mPFC connectivity in acute brain slices using electrophysiology and optogenetics. We show that the anterior cingulate cortex (ACC) is the main target of dorsal subicular projections to the mPFC, with no preference between excitatory or inhibitory neurons. In addition to superficial neurons in the ACC, the prelimbic and infralimbic PFC are also targeted by subicular fibers. Thus, these novel region- and layer-specific connections between the dSUB and the prefrontal cortices challenge existing anatomical data and refine the hippocampocortical wiring diagram.

## Highlights

• The prefrontal cortex (PFC) receives fibers from the dorsal subiculum (dSUB).
• The proximal but not the distal part of the dSUB projects to the PFC
• Mainly superficial neurons of the dorsal and ventral ACC are targeted
• Excitatory and inhibitory neurons are both optogenetically activated in the PFC

## Graphical abstract

**Figure:**
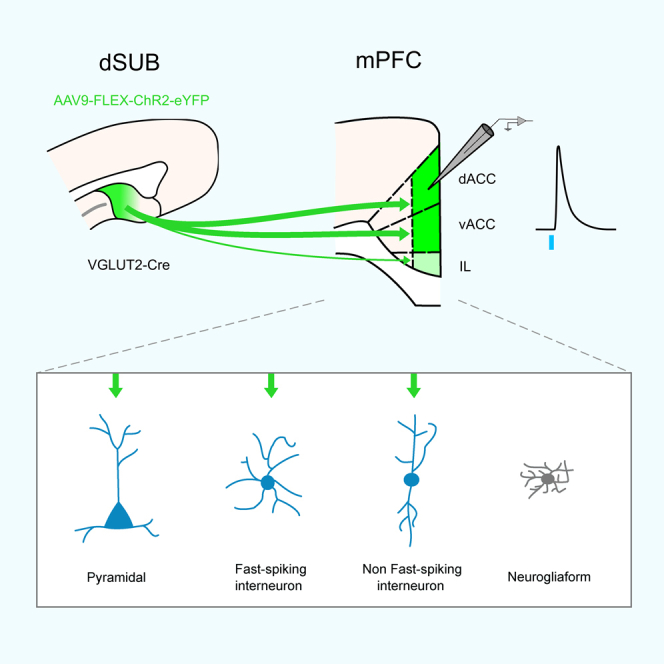

## Introduction

The hippocampal formation is an anatomically complex brain region that consists of many different subregions, including the subiculum (SUB). A dichotomy in structure and function along the longitudinal axis of the hippocampus has been proposed and supported by seminal studies (e.g. Fanselow and Dong, 2010; Floriou-Servou et al., 2018). The ventral part has been shown to be involved in emotional processes and the integration of the internal state during memory formation. Lesions of the ventral hippocampus, but not the dorsal part, for example, affect stress responses and emotional behavior. In contrast, the dorsal part facilitates cognitive processes, like navigational tasks and context coupling of memories (Strange et al., 2014).

The SUB follows the hippocampus along the longitudinal axis. It receives its main input from the CA1 area, and together with the CA1, it is considered the main hippocampal output region as it is extensively connected to various cortical and subcortical regions (e.g. Aggleton and Christiansen, 2015; Bienkowski et al., 2018). The intrinsic hippocampal neuronal circuit is maintained along the dorsoventral axis; however, a functional dichotomy has also been ascribed to the dorsal (dSUB) and ventral subiculum (vSUB). The dorsal part abundantly contains navigation-specific cells (O'Mara et al., 2009; but see Torromino et al., 2019), whereas the ventral part of the subiculum is involved in mediating stress responses (Herman and Mueller, 2006).

This dichotomy in function is also reflected in differences in gene expression and long-range connections. The dorsal hippocampus resembles cortical regions on the level of gene expression, whereas gene expression in the ventral part correlates with endocrine-involved regions, like the amygdala and the hypothalamus (Dong et al., 2009). Single-cell transcriptomic studies have further enhanced our understanding of gene expression in subicular neurons (Cembrowski et al., 2018a; Ding et al., 2020; Yao et al., 2021) highlighting different neuronal cell types in the dSUB and vSUB.

The dorsal part of the subiculum projects to cognitive regions, like the retrosplenial cortex and mammillary nuclei (Naber and Witter, 1998; Namura et al., 1994; Witter, 2006). Additionally, it also projects to the caudal part of the lateral septum and the nucleus accumbens (Fanselow and Dong, 2010; Naber and Witter, 1998). On the contrary, the ventral hippocampus is highly embedded in the limbic system of the brain projecting to the olfactory bulb, bed nucleus of the stria terminalis, amygdala (Fanselow and Dong, 2010), and thalamic and hypothalamic areas (Namura et al., 1994). Afferent fibers from the vSUB are, however, also evident in the medial prefrontal cortex (mPFC) (Jay and Witter, 1991; Liu and Carter, 2018; Naber and Witter, 1998; Witter, 2006).

The mPFC is involved in higher-order cognitive processes like attention, planning, and decision-making (Carlén, 2017). It is therefore highly connected to other cortical and subcortical regions of the brain to facilitate signal integration processes. The mPFC consists of three different subregions, the infralimbic cortex (IL), prelimbic cortex (PL), and anterior cingulate cortex (ACC), all thought to fulfill different functions and displaying different connectivity patterns (Hoover and Vertes, 2007).

Efferent fibers from the vSUB terminate in the superficial layers of the infralimbic cortex (Bienkowski et al., 2018; Naber and Witter, 1998; Wee and MacAskill, 2020). The dSUB has also been suggested to project to the deep layers of the IL and to the ACC (Bienkowski et al., 2018; Jay and Witter, 1991). However, not much is known about this projection, and data about functional connectivity are lacking.

Here, we studied the functional connectivity between the dSUB and the mPFC using channelrhodopsin-assisted circuit mapping (e.g. Liu and Carter, 2018; Petreanu et al., 2007; Wozny et al., 2018). By using this technique, we examined the location and identity of postsynaptic mPFC cells and we show that the dSUB fibers terminate throughout the mPFC but that connectivity rates are highest in the superficial layers of the anterior cingulate cortex (ACC). Light-induced synaptic responses were recorded in different types of excitatory and also inhibitory cell types including fast-spiking neurons but with the exception of putative neurogliaform cells. Our results overall suggest that the dSUB projects similarly to both inhibitory and excitatory cells potentially maintaining the excitation-inhibition balance.

## Results

### Functional connectivity between the dorsal subiculum and the prefrontal cortex

**Figure 1:**
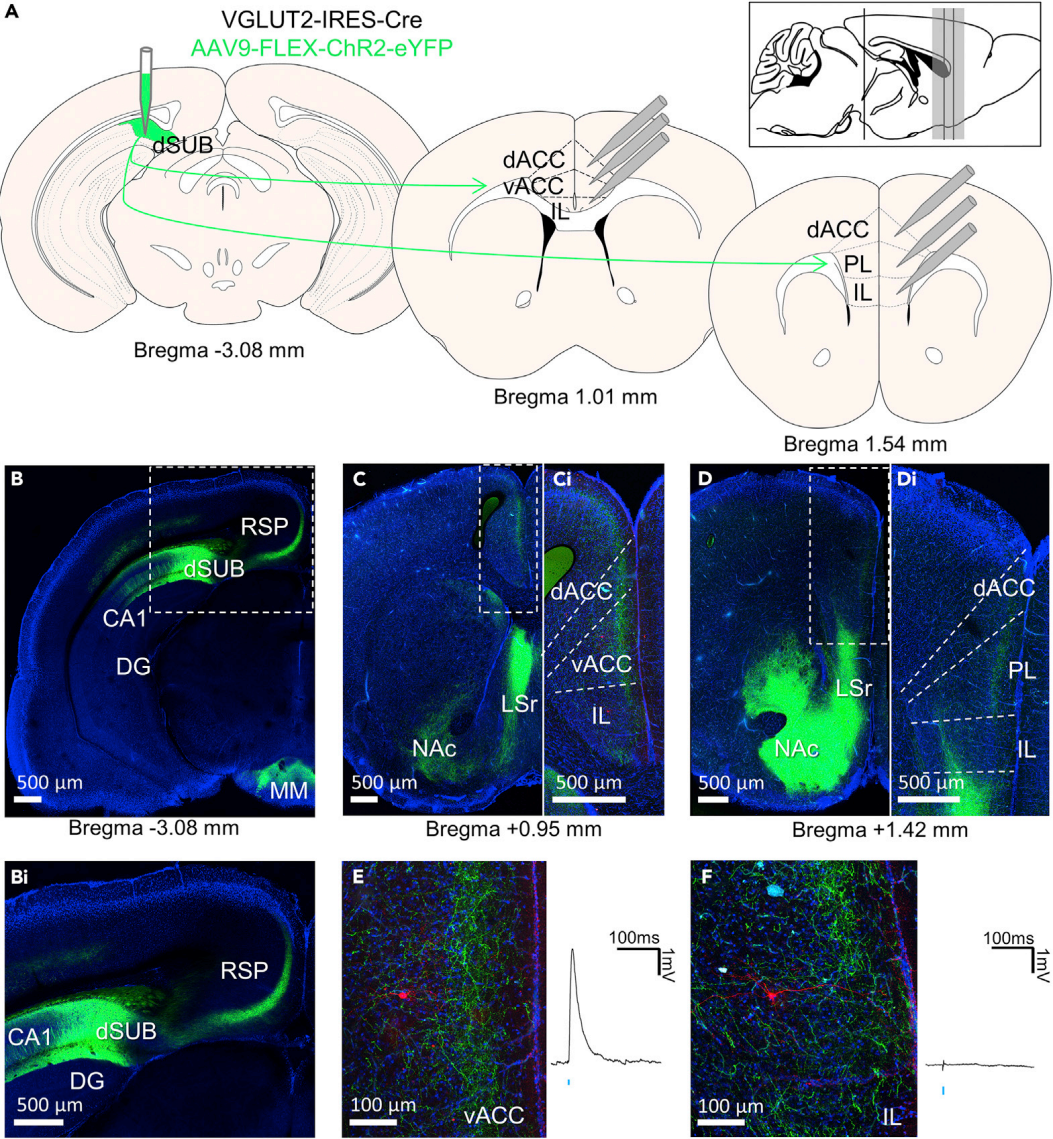
Functional connectivity between the dorsal subiculum and the prefrontal cortex (A) Overview of the experimental design. A viral vector containing ChR2 is injected into the dorsal subiculum (dSUB). Responses to light stimulation were recorded from cells in the dorsal anterior cingulate cortex (dACC), ventral ACC (vACC), prelimbic (PL) and infralimbic (IL) prefrontal cortex across different distances from bregma. (B) Image of the injection site at bregma −3.08 mm. Fibers can be seen in the retrosplenial cortex (RSP) and the medial mammillary nucleus (MM). Bi) Enlargement of the region in B. (C) Fibers in the prefrontal cortex at bregma +0.95 mm. Additionally, fibers are seen in the lateral septum (LSr) and the nucleus accumbens (NAc). Ci) Enlargement of the region in C showing fibers in the ACC, but not the IL. (D) Fibers in the prefrontal cortex at bregma +1.42 mm. Additionally, fibers can be seen in the nucleus accumbens and lateral septum. Di) Enlargement of the region in D showing fibers in the superficial layers of the PL and the deep layers of the IL. (E and F) A recorded neuron in the (E) vACC and the (F) IL on the left with the light-induced response on the right. Blue tick indicates the time point of LED stimulation. (B–D) Scale bar 500 μm. (E–F) Scale bar 100 μm. Green, eYFP; Blue, DAPI; Red, biocytin-streptavidin. All images are obtained from the same animal.

VGLUT2-IRES-Cre mice were injected with a viral vector driving expression of Cre-dependent channelrhodopsin-2 (ChR2) and enhanced yellow fluorescent protein (eYFP) into the dorsal subiculum (dSUB) to selectively target excitatory subicular neurons (Figure 1A). Since VGLUT2 is not expressed in areas surrounding the dSUB, viral expression was confined to the output neurons of the subiculum (Figure 1B) (Wozny et al., 2018). ChR2-positive fibers were observed in several different brain regions. The densest projections are visible in the retrosplenial cortex (RSP) (Kinnavane et al., 2018; Yamawaki et al., 2019), the rostral part of the lateral septum (LSr), the nucleus accumbens (NAc) and the medial mammillary nucleus (MM) (Figures 1B–1D). Additionally, slightly weaker but substantial projections are seen in the medial prefrontal cortex (mPFC), including the prelimbic cortex (PL), anterior cingulate cortex (ACC), and infralimbic cortex (IL) (Figures 1C and 1D). ChR2-positive fibers seem to be mainly confined to layer II-III for the PL and ACC (Figure 1Ci). However, fibers are only present in deep and not superficial layers of the IL in anterior parts of the mPFC (Figure 1Di).

We next investigated the functional connectivity by optogenetically stimulating VGLUT2-positive fibers that originate from the dSUB. Postsynaptic activity upon optogenetic stimulation was recorded using whole-cell patch-clamp recordings in four different regions of the mPFC: dACC, vACC, PL, and IL (Figure 1A). Light-induced excitatory postsynaptic potentials (EPSPs) were observed across multiple regions and cells confirming functional connectivity between the dSUB and the mPFC. An example of a responsive mPFC neuron is shown in Figure 1E. However, not all recorded cells responded to optogenetic stimulation (102 out of 141 recorded neurons did not and 39 did; Figure 1F), which might suggest cell or region specificity.

### Prefrontal cortex projection originates from the proximal part of the dorsal subiculum

**Figure 2:**
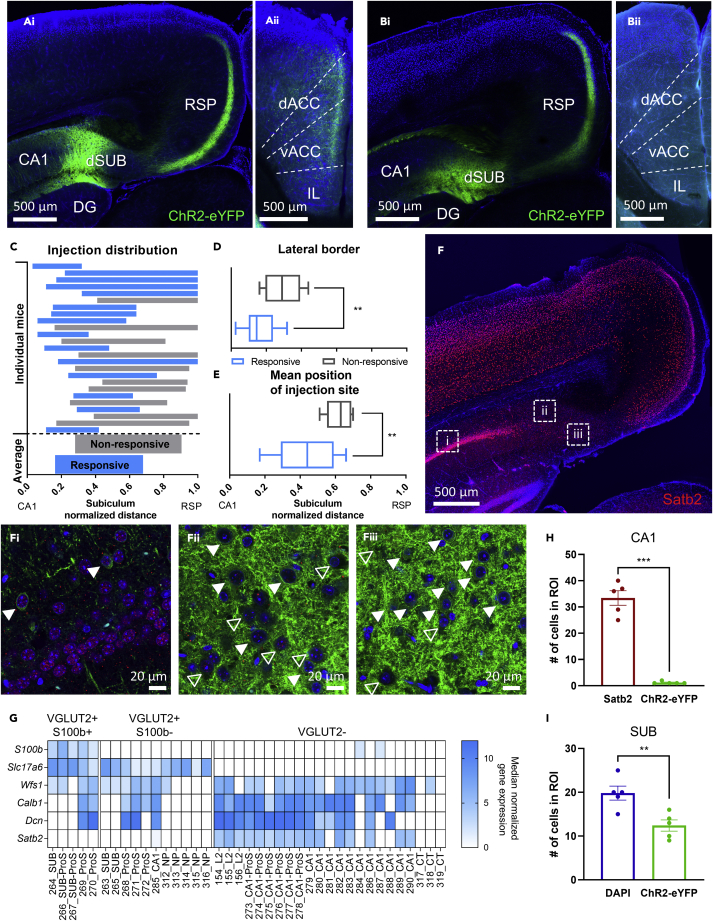
Prefrontal cortex projection originates from the proximal part of the dorsal subiculum (A and B) (Ai,Bi) Example of a (Ai) proximal and (Bi) distal injection site. Scale bar 500 μm. (Aii,Bii) Prefrontal cortex images corresponding to either (Aii) proximal or (Bii) distal injection site. Images in Ai and Bi are from the same mice as Aii and Bii, respectively. (C) Graph showing the distribution of injection sites. The upper part shows the distribution of injection sites for individual mice. Bars from mice that contained cells that were responsive to stimulation are blue, whereas bars from mice that showed no response to stimulation are gray. The bottom part of the graph displays the average injection site distribution for responsive and non-responsive mice. The width of the subiculum is normalized to 1, with the border with the CA1 and the RSP being at 0 and 1, respectively. (D) Mean normalized location of the lateral end of the injection site for responsive and non-responsive mice. (E) Mean normalized position of the injection sites for responsive and nonresponsive mice. Data in D and E are represented as boxplots with the box extending from the 25th to 75th percentiles, the middle line representing the median and the whiskers indicating the minimum and maximum values. (F) Satb2 staining of the injection site region with enlarged images of the CA1 (Fi), S100b-region of the subiculum (Fii) and middle of the subiculum (Fiii). Enlarged images show Satb2-staining in red and ChR2-eYFP expression in green. The white arrows in Fi indicate neurons that are positive for Satb2 and ChR2-eYFP. The closed arrows in Figures 2Fii & 2iii indicate ChR2-positive neurons, whereas the open arrows are ChR2-negative. (G) Gene expression in subiculum and CA1 clusters as defined and provided by the Allen Brain institute. (H) Quantification of Satb2 and ChR2-expressing cells in the CA1. Data are represented as mean ± SEM. (I) Number of DAPI cells and cells expressing ChR2 in the subiculum. Data are represented as mean ± SEM. ROI for both H and I is 184.7 × 184.7 μm in size. ∗ p<0.05, ∗∗ p<0.01, ∗∗∗ p<0.001.

Not all mice contained cells that responded to optogenetic stimulation. Prefrontal slices of these mice also seem to contain fewer fibers than the ones from mice that did respond to stimulation. With the aim to explain these differences, we compared the precise injection sites of responsive (N = 15) and nonresponsive mice (N = 10). Indeed, a subset of mice had injection sites that were centered in the proximal part of the dSUB (Figure 2Ai), whereas others showed injection sites with centers more toward the distal part (Figure 2Bi). The presence of fibers was also different for these two categories of injection sites, with fibers being present in the mPFC for proximal injections (Figure 2Aii), but rather absent for injections into the distal part of the subiculum (Figure 2Bii).

Quantifying the exact location and width of the injection site for each mouse also showed that mice that exhibited optogenetic responses had an injection site that was located closer to the CA1 than the RSP (Figure 2C). Both the lateral border of the injection site and the average position of the injection site was located more toward the CA1 for responsive mice compared with nonresponsive mice (lateral border: t test, p = 0.0019; mean position: t test, p = 0.0016; Figures 2D and 2E). These data, together with previous literature, suggest that prefrontal-projecting neurons are located in the proximal part of the dorsal subiculum (Bienkowski et al., 2018; Cembrowski et al, 2018a, 2018b; Kim and Spruston, 2012). Therefore, only the data from proximal dSUB-injected mice were analyzed further. However, relative injection sites are overlapping between individual responsive and nonresponsive mice (Figure 2C) and therefore an exact location of prefrontal-projecting neurons cannot be derived from these data.

To demonstrate that CA1 neurons are not infected by viral vectors, we stained against *Satb2* to specifically label CA1 neurons but not VGLUT2-expressing subicular output neurons. *Satb2* expression was shown to be indeed restricted to the CA1 region and the retrosplenial cortex, providing exact borders of the subiculum (Figure 2F). Very few cells in the CA1 region showed coexpression of *Satb2* and ChR2-eYFP (Figure 2H). Of note, these cells were found outside the pyramidal layer of CA1 indicating that these might rather be subicular neurons as the distal CA1 and proximal SUB have been shown to overlap (Figure 2Fi). To further validate our data, we chose to explore coexpression pattern of additional markers such as *S100b*, *Slc17a6* (encoding for VGLUT2), *Wfs1*, *Calb1*, *Dcn,* and *Satb2* across different cell types of CA1 and subicular neurons by using publicly available single-cell RNA sequencing (scRNAseq) data (Tasic et al., 2018). In line with previous reports (Cembrowski et al., 2018a), VGLUT2-positive neurons could be segregated into *S100b*-positive and *S100b*-negative neurons. *Wfs1*, *Calb1* and *Dcn* were enriched in both VGLUT2-positive and VGLUT2-negative in the CA1 region, as well as the SUB (Figure 2G) rendering them useless for our purpose. However, only VGLUT2-negative neurons contain considerable mRNA levels for *Satb2* (Figures 2F and 2G). This further supports our finding that ChR2-eYFP expression is limited to the dSUB and hardly seen in *Satb2*-positive neurons, if at all (see Figures 2Fiii and 2I).

### Proximal dorsal subiculum neurons project preferably to superficial layers of the anterior cingulate cortex in the posterior part of the prefrontal cortex

**Figure 3:**
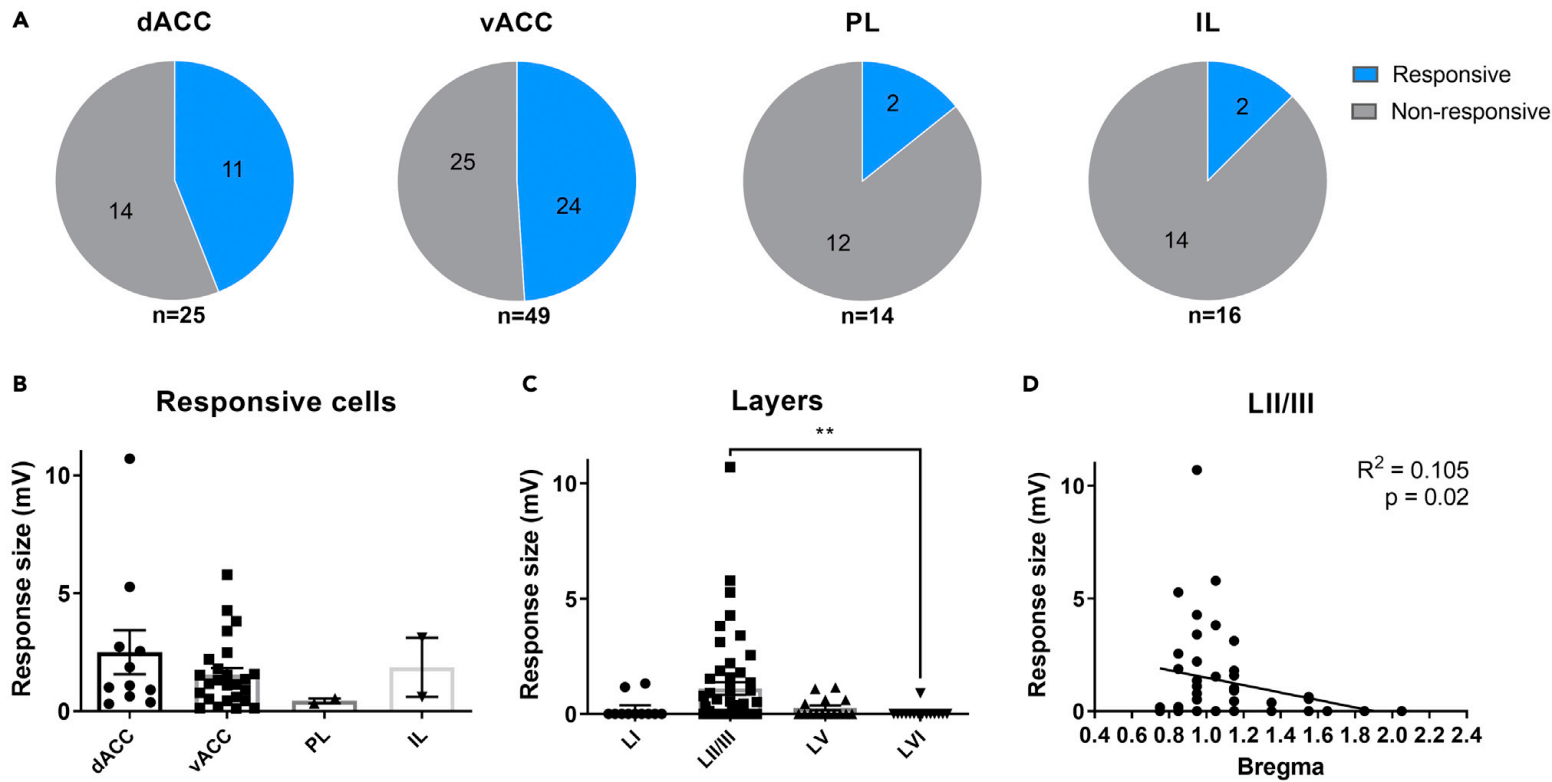
Proximal dorsal subiculum neurons project preferably to superficial layers of the anterior cingulate cortex in the posterior part of the prefrontal cortex (A) Proportion of recorded cells that were responsive or non-responsive to light stimulation in the dACC, vACC, PL and IL. (B) Response size in responsive cells recorded from different regions of the mPFC. Data are represented as mean ± SEM. (C) Response size of all neurons per layer. LI, n = 11; LII/III, n = 51; LV, n = 15; LVI, n = 15. Data are represented as mean ± SEM. (D) Correlation between response size and distance from bregma for LII/III neurons (n = 51). ∗ p<0.05, ∗∗ p<0.01, ∗∗∗ p<0.001.

Neurons responsive to optogenetic stimulation (39 out of 103 neurons) were found across all four regions of the mPFC. However, the proportion of responsive cells differed significantly (Chi-square test, p = 0.01; Figure 3A). The vACC had a higher proportion of responses than the IL and PL (Fisher's exact test, vACC vs IL, p = 0.017; vACC vs PL, p = 0.030), but there was no significant difference between the vACC and the dACC (Fisher's exact test, p > 0.999). This indicates that the subiculum has stronger projections to the ACC than other regions of the mPFC. However, the response sizes (EPSPs) do not differ between the subregions of the mPFC (Figure 3B). Nonetheless, it must be noted that the numbers of recorded responses are low for the IL (n = 2) and PL (n = 2).

Next, the exact location of recorded neurons was determined to get a more precise idea of where the subicular neurons are projecting to in the mPFC. The position of each postsynaptic neuron was determined in relation to cortical layers and PFC regions. Dividing all recorded neurons among cortical layers revealed that response size is not uniform across layers (Kruskal-Wallis test, p = 0.007; Figure 3C). Post hoc comparison only revealed a significant difference of LII/III with LVI (p = 0.005), but no significant differences with LI (p = 0.139) and LV (p = 0.357). Since LII/III harbors the most responsive neurons, linear regression between response size and bregma for specifically LII/III neurons was performed. This revealed a correlation, with higher responses being present in the posterior PFC (R^2^ = 0.105, p = 0.02; Figure 3D).

Together, this indicates that the subiculum projects more strongly to superficial layers in more dorsal regions and posterior regions of the mPFC. The anatomy of the mPFC differs between anterior and posterior parts. The PL is present in the anterior mPFC, but this is being replaced by the vACC in the posterior part. This shift happens around 1.42 mm from bregma. This change is also visible in our data, with barely any responses present further away from bregma and a rapid increase in response rate closer than 1.42 mm to bregma (Figure 3D).

### Proximal dorsal subiculum drives activity of both inhibitory and excitatory neurons in the mPFC

**Figure 4:**
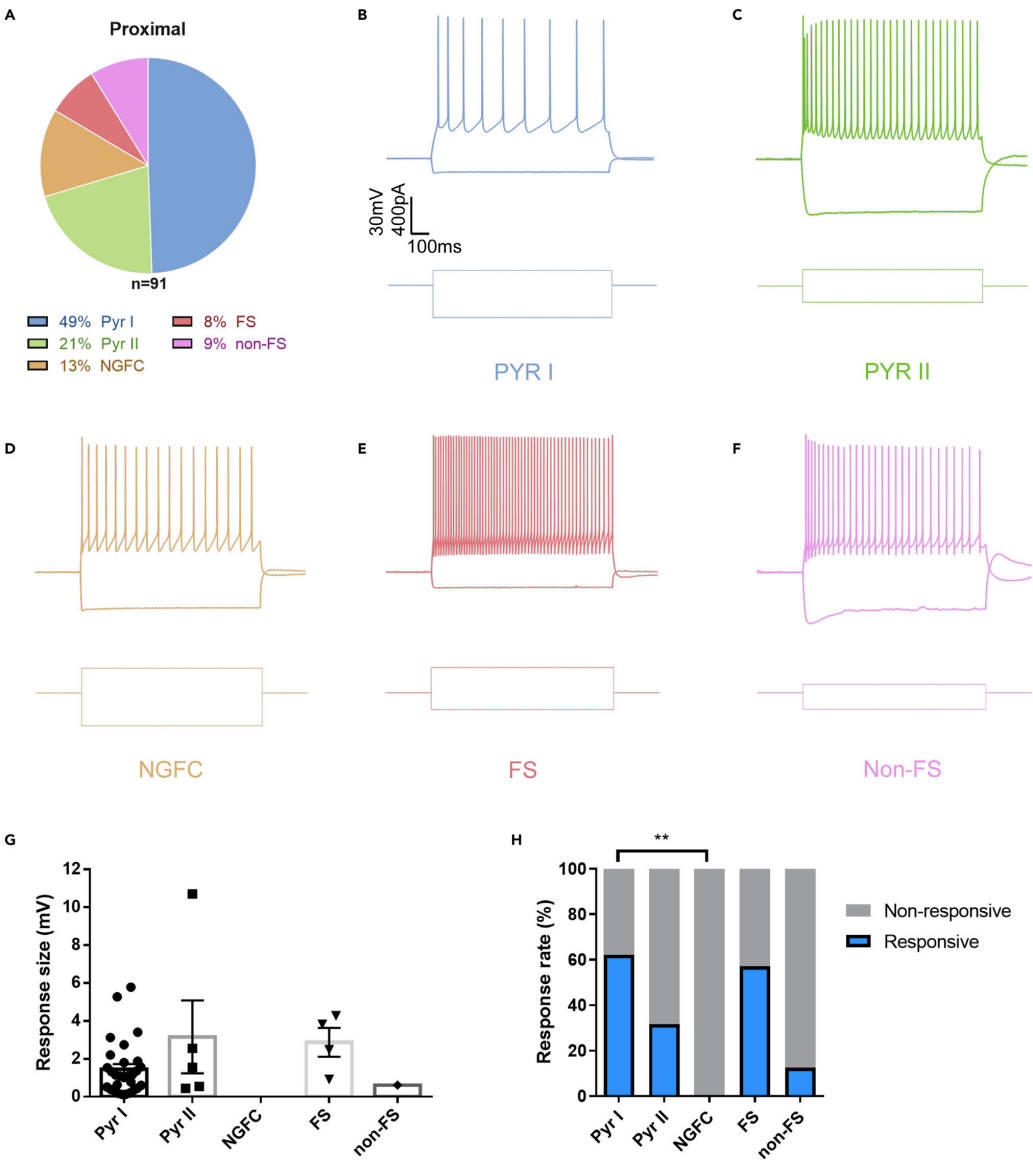
Proximal dorsal subiculum drives activity of both inhibitory and excitatory neurons in the mPFC (A) Proportion of cell types recorded in layer II-III of proximally injected mice. (B–F) Spiking pattern and hyperpolarization in response to current injection for (B) pyramidal type I cells, (C) pyramidal type II cells, (D) neurogliaform cells, (E) fast spiking interneurons and (F) non-fast spiking interneurons. (G) Response size upon light stimulation per cell type. Data are represented as mean ± SEM. (H) Proportion of cells responsive to optogenetic stimulation per cell type. Pyr I, n = 45; Pyr II, n = 19; NGFC, n = 12; FS, n = 7; non-FS, n = 8. ∗ p<0.05, ∗∗ p<0.01, ∗∗∗ p<0.001.

Responses were recorded from a few different cell types in layer II–III, both from excitatory and putative inhibitory neurons (Figure 4A). Cells were classified based on their firing pattern, hyperpolarization and morphology, resulting in four categories: pyramidal cells (n = 64), neurogliaform cells (n = 12), fast-spiking interneurons (n = 7), and putative nonfast spiking interneurons (n = 8) (Figures 4B–4F). Neurogliaform cells showed the well-described characteristic late-spiking phenotype (e.g. Kawaguchi, 1995; Schuman et al., 2019), whereas all interneurons that did not fulfill the criteria of a fast-spiking or a late-spiking cell were classified as non-fast-spiking (putative) interneurons.

Two types of pyramidal cells were identified using clustering analysis on recordings of pyramidal cells from all mice, both responsive and nonresponsive. Type II (n = 30) pyramidal cells often did not display a clear sag potential and had a higher firing frequency upon current injection than type I (n = 46) (Figures 4B and 4C; see also Figure S1). The two types of pyramidal cells differed in electrophysiological characteristics (two-way ANOVA, p < 0.001), but showed no clear differences in morphology (Figures S1B and S1C). Type II pyramidal cells have a higher input resistance (one-way ANOVA followed by Bonferroni's multiple comparison test, p < 0.0001; Figure S1G), but the cell types did not differ in RMP (p = 0.7767; Figure S1F). The access resistance did not cause the difference in input resistance since this was not significantly different (p > 0.999; Figure S1H). Additionally, type II pyramidal neurons were firing at higher frequencies than type I cells at the same current injections, therefore exhibiting higher excitability than type I pyramidal cells (two-way ANOVA, p < 0.001; Figures S1D and S1E).

The response size upon optogenetic stimulation did not differ between cell types (Figure 4G). However, the proportion of responses was not uniform for the different cell types (Chi-square test, p < 0.0001; Figure 4H). The response rate differs significantly between Pyr I and NGFC (p = 0.001). Since NGFCs are mainly located superficially in LI to LIII and have stubby dendrites, this could explain why none of the 12 recorded cells exhibited responses to optogenetic stimulation. On the other hand, there were no differences between Pyr I, Pyr II, FS, and non-FS neurons. Therefore, the dSUB projects to both excitatory and inhibitory neurons in the mPFC.

## Discussion

We have investigated and determined neuronal connections of VGLUT2-positive neurons of the dSUB to specific subregions and different cell types in the PFC. We found that proximal dSUB neurons mainly project to the superficial layers of both the dACC and vACC, but neuronal responses were also recorded in the IL and PL neurons following optogenetic stimulation. Interestingly, both excitatory and inhibitory neurons were innervated, except for neurogliaform cells. Our detailed analysis of the viral injection sites revealed that dSUB neurons in the proximal part are more likely to project to the mPFC than neurons in the distal part of the dSUB (Figure 2). However, in a few mice, we observed infected neurons in the proximal part, without finding responsive neurons nor fluorescently labeled fibers in the mPFC. This suggests that connectivity patterns may be more variable, but the exact reason for that remains unclear.

Powerful transcriptomics analyses have recently refined the grouping of subicular neurons (Cembrowski et al, 2018a, 2018b; Ding et al., 2020; Tasic et al., 2018). Classical electrophysiological studies identified two major classes of pyramidal neurons (Staff et al., 2000); however, in combination with retrograde tracing methods spatially clustered neurons have indicated more than these two major classes (Kim and Spruston, 2012). Different subregions of the SUB, the proximal and distal part of either the dSUB, but also the vSUB, may comprise up to over 20 different classes of neurons (Cembrowski et al, 2018a, 2018b; Ding et al., 2020).

Cembrowski et al demonstrated that 5 out of 8 identified groups of subicular pyramidal neurons project to the PFC expressing the following molecular markers: *S100b*, *Dlk1*, *Tpbg*, *Gpc3*, and *Cbln4*, respectively. *S100b* expression is enriched in dSUB neurons, whereas *Gpc3* expression is more abundant ventrally. *Dlk1*, *Tpbg,* and *Cbln4* are expressed along the entire dorsoventral axis. The vesicular glutamate transporter 2, short VGLUT2 *(encoded by Slc17a6)*, however, is only been seen in *S100b* neurons (apart from *Fn1* and *Col5a2* that do not project to the PFC) (Cembrowski et al., 2018a).

We therefore decided to utilize VGLUT2-Cre mice to investigate the connectivity between dSUB and prefrontal neurons. The S100b-Cre mouse line might have been useful to study these connections; however, *S100b* seems to be expressed in other hippocampal neurons (Tanaka et al., 2008), whereas VGLUT2 is not expressed in adjacent regions to the SUB such as the CA1 area making it a versatile tool to study dSUB output (Wozny et al., 2018).

In line with previous data for CA1 projections, we further found a gradient in subicular-cortical connectivity (Ferreira-Fernandes et al., 2019). Ferreira-Fernandes et al showed an increase in connectivity strength from the ACC to RSP implying that synaptic responses increase along the anterior-posterior axis of the brain. We found the same for dSUB to the mPFC, with an increase in synaptic strength more posterior in the brain (Figure 3). However, we only tested the PFC as data for the RSP suggest a robust connection (Kinnavane et al., 2018; Nitzan et al., 2020; Yamawaki et al., 2019).

The similarity in synaptic strength along the anterior-posterior axis seems surprising; however, one might speculate that the signal that arises from SUB neurons may function as an amplification signal. Hippocampal CA1 neurons project both to the ACC and the SUB (Ferreira-Fernandes et al., 2019). SUB projection neurons similarly target ACC neurons to amplify the hippocampal signal that they received. Future optoelectrophysiological studies using two color optogenetic stimulation (Hooks, 2018) may therefore investigate whether dCA1 and dSUB neurons convergently target neurons located in the ACC and RSP.

In addition, we further found no preference of dSUB fibers targeting prefrontal neurons. Both inhibitory interneurons and excitatory pyramidal neurons respond upon input stimulation. This scenario may suggest that there is a tight balance of excitatory and inhibitory tone. Intriguingly, such balanced networks have been proposed to provide a highly efficient way of neuronal coding and information processing (Denève and Machens, 2016).

### Limitations of the study

Given the high number of different classes of neurons, both in the dSUB, as well as in the downstream target regions, the mPFC, our study may pave the way for more refined analyses taking novel transgenic animal models in terms of their cell identity and their additional target regions into account. Combining these sophisticated methods will be key to unravel the details of the connectivity between the main hippocampal out region and the prefrontal cortex.

## STAR★Methods

### Key resources table

**Table:** 

REAGENT or RESOURCE	SOURCE	IDENTIFIER
**Antibodies**		

Streptavidin Alexa-647	Life Technologies	Cat# S32357
Rabbit anti-Satb2	Abcam	Cat# Ab92446; RRID: AB_10563678
Goat anti-Rabbit Alexa-647	Abcam	Cat# A21245; RRID: AB_2535813

**Bacterial and virus strains**		

pAAV-EF1α-Switch:NLSmRuby2/ChR2(H134R)-EYFP-HGHpA	Viral Core Facility, Charité (now available from Addgene: Plasmid #118279)	

**Experimental models: Organisms/strains**		

Mouse: Slc17a6^tm2(cre)Lowl^	Jackson Laboratory	016963

**Software and algorithms**		

Prism	GraphPad Software	Version 8
Axograph X	Axograph Scientific	Version 1.7.6
ClustVis	Metsalu and Vilo (2015)	N/A
Fiji (ImageJ)	https://fiji.sc/	N/A

**Other**		

Multiclamp 700B Amplifier	Molecular Devices	
Blue LED pE-300^Ultra^	Cool LED	
Vibratome	Leica Biosystems	
Fluoromount-G with DAPI	ThermoFischer Scientific	00-4959
Whole cortex & hippocampus – 10x genomics (2020) with 10x-smart-seq taxonomy (2020)	Allen Brain Institute https://portal.brain-map.org/atlases-and-data/rnaseq	

### Resource availability

#### Lead contact

Further information and requests for resources should be directed to and will be fulfilled by the Lead Contact, Christian Wozny (christian.wozny@medicalschool-hamburg.de).

#### Materials availability

This study did not generate new unique reagents.

#### Data and code availability

All data supporting the findings of this study are given in the main text or the supplemental information. This study did not generate a novel program code.

### Experimental model and subject details

#### Animals

Both male and female VGLUT2-IRES-Cre mice Slc17a6^tm2(cre)Lowl^ (JAX stock #016963, The Jackson Laboratory; Vong et al., 2011) on a C57BL/6 background were used in this study. Mice were kept on a regular 12:12 hour light/dark cycle with ad libitum access to water and food. Pups were housed with their parents until weaning at P21. All animal procedures were approved by the Ethics committee of the University of Strathclyde, Glasgow in accordance with the relevant UK legislation (the Animals (Scientific Procedures) Act, 1986).

### Method details

#### Viral injections

Mice (N=25) underwent stereotaxic surgery at P27-P33. They were deeply anaesthetized using isoflurane (5% for induction; 1-2% for maintenance), transferred to a stereotaxic frame (Narishige, Tokyo, Japan) and were subcutaneously injected with the analgesics carprofen (5 mg/kg) subcutaneous and lidocaine (4 mg/kg) under the scalp. A small incision was made and the skull was exposed under aseptic conditions. Bregma and lambda were measured, and the head tilt was adjusted accordingly to ensure that the brain was level. A small burr hole was drilled above the subiculum in the left hemisphere. Glass micropipettes were pulled using a PC-100 vertical puller (Narishige, Tokyo, Japan). Injection coordinates that were used are (AP -3.40, ML -2.00, DV -1.80 mm) for the medial subiculum and (AP -3.40, ML -2.50, DV -1.80 mm) for the lateral subiculum, relative to bregma. Injections were made using a pressure injector (Narishige, Tokyo, Japan), injecting 200 nL of virus at a rate of 25 nL/min. The viral vector used is a pAAV-EF1α-Switch:NLSmRuby2/ChR2(H134R)-EYFP-HGHpA (titer: 2 x 10^13^ vector genomes/mL) which is an adeno-associated virus of serotype 9 that drives Cre-dependent expression of ChR2 and YFP in Cre-positive cells and the expression of mRuby2 in the absence of Cre (Wozny et al., 2018). The pipette containing the viral vectors was left in place for 10 minutes after injection before slow removal to minimize viral spread along the injection tract. The burr hole was covered with bone wax and the incision was closed using Vetbond tissue adhesive. Mice were given at least three weeks after surgery before slice preparation to allow sufficient viral expression.

#### Acute slice preparation

Mice were humanely euthanized using cervical dislocation and immediate decapitation. Brains were quickly removed and submerged in oxygenated (95% O_2_; 5% CO_2_) ice-cold sucrose-containing artificial cerebrospinal fluid (ACSF) containing (in mM): sucrose 50, NaCl 87, NaHCO3 25, KCl 3, NaH_2_PO_4_ 1.25, CaCl_2_ 0.5, MgCl_2_ 3, sodium pyruvate 3 and glucose 10. Brains were kept in the dark after extraction until the end of the experiment. Brains were sliced in 300 μm coronal slices using a vibratome (Leica Biosystems, Newcastle-upon-Tyne, UK). Prefrontal cortex slices were transferred to oxygenated ACSF containing sucrose at 35°C and after 30 min incubation transferred to 35°C normal ACSF containing (in mM) NaCl 115, NaHCO_3_ 25, KCl 3, NaH_2_PO_4_ 1.25, CaCl_2_ 2, MgCl_2_ 1, sodium pyruvate 3 and glucose 10. Slices were left to cool down to room temperature (∼ 30 min) before recordings. The rest of the brain slices were directly transferred to 4% PFA in 0.1 M sodium-based phosphate-buffered saline (PBS) overnight and stored in 0.1 M PBS until further tissue processing.

#### Electrophysiology

For electrophysiological recording, slices were transferred to a recording chamber and continuously perfused with oxygenated ACSF at a flow rate of 2-3 ml/min. Slices were visualized using a Luigs and Neumann LN-Scope System (Luigs and Neumann, Ratingen, Germany). The recording location was determined using a 4x objective, and cell patching was performed under a 60x objective. Cells were recorded in the prefrontal cortex, spanning the infralimbic cortex (IL), prelimbic cortex (PL) and the dorsal and ventral parts of the anterior cingulate cortex (ACC) using the MultiClamp 700B Amplifier (Molecular Devices, California, USA). Glass micropipettes were filled with an intracellular solution containing (in mM) potassium gluconate 125, hepes 10, KCl 6, EGTA 0.2, MgCl_2_ 2, Na-ATP 2, Na-GTP 0.5, sodium phosphocreatine 5, and 0.2 % biocytin. The pH of the intracellular solution was adjusted to 7.2 by adding KOH. Whole-cell patch clamping was performed, and cells were held at -60 mV in voltage-clamp to assess access quality and series resistance. Electrical properties were recorded in current-clamp using a 1 s current injection with increasing amplitude (range -500–500 pA; step size 10–100 pA). Resting membrane potential (RMP) was determined using multiple sweeps of 500ms without current injection and input resistance was measured by injecting a 1s pulse of -100 pA.

Optogenetic stimulation was performed using a blue LED (pE-300^ultra^, Cool LED, Andover, UK; 2 ms pulse; power 11.5 mW) and responses were recorded in current clamp. Response size was determined as an average of 20 stimulations. Recordings were performed in darkness to prevent unintentional stimulation. All neuronal voltage and current signals were low pass-filtered at 2-10 kHz and acquired at 10-25 kHz using an ITC-18 digitizer interface (HEKA, Pfalz, Germany).

#### Post hoc staining

Following electrophysiological recordings, slices containing neurons which had been patched and filled with biocytin were processed as previously described (Wozny and Williams, 2011). Slices were blocked for 1.5 hours using 5% normal goat serum and 1% Triton X-100 dissolved in 0.1M PBS. Slices were incubated with streptavidin conjugated to Alexa-647 (1:500 dilution; Life Technologies, Paisley, UK) in 0.1M PBS containing 1% Triton-X for 3 hours at room temperature. After incubation, slices were washed with PBS and mounted using DAPI-containing Vectashield mounting medium (Vector Labs, Peterborough, UK) or Fluoromount-G mounting medium (ThermoFisher Scientific, Waltham, MA, USA).

Injection site slices of mice (N=5) were stained for Satb2. Slices were blocked for 2 hours using 5% normal goat serum and 1% Triton X-100 dissolved in 0.1M PBS. Slices were incubated with primary antibody rabbit anti-Satb2 (1:1000, Identifier Cat#: ab92446, Abcam, Cambridge, UK) overnight. The next day, slices were treated with PBS, Triton-X and a secondary antibody (Alexa-647, 1:500; Identifier Cat#: A21245) as described above.

Other non-recorded slices were washed with 0.1M PBS and directly mounted with DAPI-containing Vectashield or Fluoromount-G mounting medium.

#### Imaging

All images were taken using a Leica SP8 confocal microscope. Tile scanning was used to create overview images, and stitching was done automatically by the Leica LASX software. Images were taken using a 10x objective. Z-stacks of prefrontal cortex slices were made with 5-8 μm steps with 6-8 steps for each stack in order to reconstruct recorded neurons and to visualize ChR2-positive fibers.

### Quantification and statistical analysis

Electrophysiological recordings were analyzed using AxographX. Response size of optogenetic stimulation was measured by subtracting the baseline and then measuring the peak amplitude in the second of the trace following the LED pulse. Neurons were classified by action potential firing patterns and cell morphology using post-hoc staining. Type I and type II pyramidal neurons were separated by clustering analysis using ClustVis with Euclidean distance as the similarity measure and hierarchical clustering with average linkage (Metsalu and Vilo, 2015). Patched neurons with damaged dendrites due to tissue sectioning were excluded from data analysis.

Confocal images were analyzed using ImageJ. The distribution and spread of the injection site were measured relative to the length of the subiculum since the size of the subiculum is different along the anterior-posterior axis. The distance from the CA1 to one side of the injection site and the distance from the RSP to the other side of the injection site was divided by the total length of the subiculum, as measured by drawing a straight line from the CA1 to the RSP.

Median clustered gene expression data was downloaded from the Allen Brain institute (https://portal.brain-map.org/atlases-and-data/rnaseq; whole cortex & hippocampus – 10x genomics (2020) with 10x-smart-seq taxonomy (2020)). MATLAB was used to extract genes and clusters of interest from the data matrix.

Statistical analysis was performed in GraphPad Prism. Graphs were generated in GraphPad Prism. Statistical tests used were one-way ANOVA or two-way ANOVA followed by post hoc Bonferroni multiple comparison test, two-tailed unpaired t-test, linear regression, or Fishers’ exact test. The thresholds for statistical significance were: ∗p < 0.05; ∗∗p < 0.01; and ∗∗∗p < 0.001.

## Footnotes

Supplemental information can be found online at https://doi.org/10.1016/j.isci.2021.102856.

## References

[bib1] Aggleton J.P., Christiansen K. (2015). The subiculum: the heart of the extended hippocampal system. Prog. Brain Res..

[bib2] Bienkowski M.S., Bowman I., Song M.Y., Gou L., Ard T., Cotter K., Zhu M., Benavidez N.L., Yamashita S., Abu-Jaber J. (2018). Integration of gene expression and brain-wide connectivity reveals the multiscale organization of mouse hippocampal networks. Nat. Neurosci..

[bib3] Carlén M. (2017). What constitutes the prefrontal cortex?. Science.

[bib4] Cembrowski M.S., Wang L., Lemire A.L., Copeland M., DiLisio S.F., Clements J., Spruston N. (2018). The subiculum is a patchwork of discrete subregions. Elife.

[bib5] Cembrowski M.S., Phillips M.G., DiLisio S.F., Shields B.C., Winnubst J., Chandrashekar J., Bas E., Spruston N. (2018). Dissociable structural and functional hippocampal outputs via distinct subiculum cell classes. Cell.

[bib6] Denève S., Machens C.K. (2016). Efficient codes and balanced networks. Nat. Neurosci..

[bib7] Ding S.-L., Yao Z., Hirokawa K.E., Nguyen T.N., Graybuck L.T., Fong O., Bohn P., Ngo K., Smith K.A., Koch C. (2020). Distinct transcriptomic cell types and neural circuits of the subiculum and prosubiculum along the dorsal-ventral Axis. Cell Rep..

[bib8] Dong H.-W., Swanson L.W., Chen L., Fanselow M.S., Toga A.W. (2009). Genomic-anatomic evidence for distinct functional domains in hippocampal field CA1. Proc. Natl. Acad. Sci. U S A.

[bib9] Fanselow M.S., Dong H.-W. (2010). Are the dorsal and ventral Hippocampus functionally distinct structures?. Neuron.

[bib10] Ferreira-Fernandes E., Pinto-Correia B., Quintino C., Remondes M. (2019). A gradient of hippocampal inputs to the medial mesocortex. Cell Rep..

[bib11] Floriou-Servou A., von Ziegler L., Stalder L., Sturman O., Privitera M., Rassi A., Cremonesi A., Thöny B., Bohacek J. (2018). Distinct proteomic, transcriptomic, and epigenetic stress responses in dorsal and ventral Hippocampus. Biol. Psychiatry.

[bib12] Herman J.P., Mueller N.K. (2006). Role of the ventral subiculum in stress integration. Behav. Brain Res..

[bib13] Hooks B.M. (2018). Dual-channel photostimulation for independent excitation of two populations. Curr. Protoc. Neurosci..

[bib14] Hoover W.B., Vertes R.P. (2007). Anatomical analysis of afferent projections to the medial prefrontal cortex in the rat. Brain Struct. Funct..

[bib15] Jay T.M., Witter M.P. (1991). Distribution of hippocampal CA1 and subicular efferents in the prefrontal cortex of the rat studied by means of anterograde transport of Phaseolus vulgaris-leucoagglutinin. J. Comp. Neurol..

[bib16] Kawaguchi Y. (1995). Physiological subgroups of nonpyramidal cells with specific morphological characteristics in layer II/III of rat frontal cortex. J. Neurosci..

[bib17] Kim Y., Spruston N. (2012). Target-specific output patterns are predicted by the distribution of regular-spiking and bursting pyramidal neurons in the subiculum. Hippocampus.

[bib18] Kinnavane L., Vann S.D., Nelson A.J.D., O’Mara S.M., Aggleton J.P. (2018). Collateral projections innervate the mammillary bodies and retrosplenial cortex: a new category of hippocampal cells. ENeuro.

[bib19] Liu X., Carter A.G. (2018). Ventral hippocampal inputs preferentially drive corticocortical neurons in the infralimbic prefrontal cortex. J. Neurosci..

[bib20] Metsalu T., Vilo J. (2015). ClustVis: a web tool for visualizing clustering of multivariate data using Principal Component Analysis and heatmap. Nucleic Acids Res..

[bib21] Naber P.A., Witter M.P. (1998). Subicular efferents are organized mostly as parallel projections: a double-labeling, retrograde-tracing study in the rat. J. Comp. Neurol..

[bib22] Namura S., Takada M., Kikuchi H., Mizuno N. (1994). Topographical organization of subicular neurons projecting to subcortical regions. Brain Res. Bull..

[bib23] Nitzan N., McKenzie S., Beed P., English D.F., Oldani S., Tukker J.J., Buzsáki G., Schmitz D. (2020). Propagation of hippocampal ripples to the neocortex by way of a subiculum-retrosplenial pathway. Nat. Commun..

[bib24] O’Mara S.M., Sanchez-Vives M.V., Brotons-Mas J.R., O’Hare E. (2009). Roles for the subiculum in spatial information processing, memory, motivation and the temporal control of behaviour. Prog. Neuro Psychopharmacol. Biol. Psychiatry.

[bib25] Petreanu L., Huber D., Sobczyk A., Svoboda K. (2007). Channelrhodopsin-2–assisted circuit mapping of long-range callosal projections. Nat. Neurosci..

[bib26] Schuman B., Machold R.P., Hashikawa Y., Fuzik J., Fishell G.J., Rudy B. (2019). Four unique interneuron populations reside in neocortical layer 1. J. Neurosci..

[bib38] Staff N.P., Jung H.Y., Thiagarajan T., Yao M., Spruston N. (2000). Resting and active properties of pyramidal neurons in subiculum and CA1 of rat hippocampus. J. Neurophysiol..

[bib27] Strange B.A., Witter M.P., Lein E.S., Moser E.I. (2014). Functional organization of the hippocampal longitudinal axis. Nat. Rev. Neurosci..

[bib28] Tanaka M., Yamaguchi K., Tatsukawa T., Nishioka C., Nishiyama H., Theis M., Willecke K., Itohara S. (2008). Lack of connexin43-mediated Bergmann glial gap junctional coupling does not affect cerebellar long-term depression, motor coordination, or eyeblink conditioning. Front. Behav. Neurosci..

[bib29] Tasic B., Yao Z., Graybuck L.T., Smith K.A., Nguyen T.N., Bertagnolli D., Goldy J., Garren E., Economo M.N., Viswanathan S. (2018). Shared and distinct transcriptomic cell types across neocortical areas. Nature.

[bib30] Torromino G., Autore L., Khalil V., Mastrorilli V., Griguoli M., Pignataro A., Centofante E., Biasini G.M., De Turris V., Ammassari-Teule M. (2019). Offline ventral subiculum-ventral striatum serial communication is required for spatial memory consolidation. Nat. Commun..

[bib31] Vong L., Ye C., Yang Z., Choi B., Chua S., Lowell B.B. (2011). Leptin action on GABAergic neurons prevents obesity and reduces inhibitory tone to POMC neurons. Neuron.

[bib32] Wee R.W.S., MacAskill A.F. (2020). Biased connectivity of brain-wide inputs to ventral subiculum output neurons. Cell Rep..

[bib33] Witter M.P. (2006). Connections of the subiculum of the rat: topography in relation to columnar and laminar organization. Behav. Brain Res..

[bib34] Wozny C., Williams S.R. (2011). Specificity of synaptic connectivity between layer 1 inhibitory interneurons and layer 2/3 pyramidal neurons in the rat neocortex. Cereb. Cortex.

[bib35] Wozny C., Beed P., Nitzan N., Pössnecker Y., Rost B.R., Schmitz D. (2018). VGLUT2 functions as a differential marker for hippocampal output neurons. Front. Cell. Neurosci..

[bib36] Yamawaki N., Corcoran K.A., Guedea A.L., Shepherd G.M.G., Radulovic J. (2019). Differential contributions of glutamatergic Hippocampal→Retrosplenial cortical projections to the formation and persistence of context memories. Cereb. Cortex.

[bib37] Yao Z., van Velthoven C.T.J., Nguyen T.N., Goldy J., Sedeno-Cortes A.E., Baftizadeh F., Bertagnolli D., Casper T., Chiang M., Crichton K. (2021). A taxonomy of transcriptomic cell types across the isocortex and hippocampal formation. Cell.

