# Supplementary material for: Probing subicular inputs to the medial prefrontal cortex
Source: iScience. 2021 Jul 10;24(8):102856. doi: 10.1016/j.isci.2021.102856 (PMC8333156; doi:10.1016/j.isci.2021.102856)
Supplement: Document S1. Figure S1 [file mmc1.pdf]

iScience, Volume *24*

Supplemental information

Probing subicular inputs to the medial prefrontal cortex

Sanne Beerens, Rozan Vroman, Jack F. Webster, and Christian Wozny

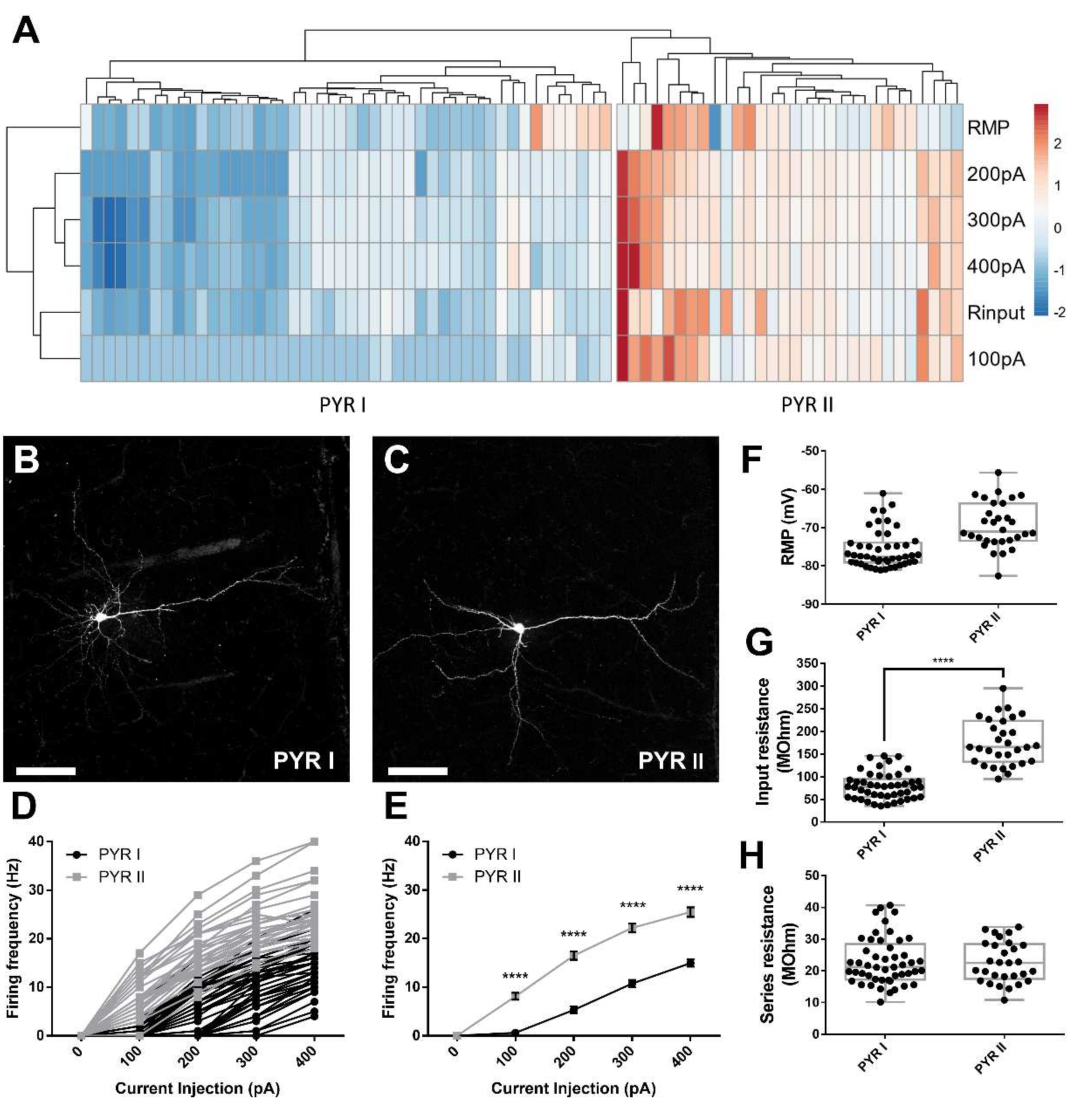

Figure S1. PYR II cells have higher excitability than PYR I cells. Related to Figure 4.

A) Heatmap generated through ClustVis showing clustering of type I and type II pyramidal cells based on RMP, input resistance and firing frequency at current injections of 100-400 pA. Settings used are Euclidean distance as the similarity measure and hierarchical clustering with average linkage. **B-C**) Example Z-projection confocal images of a (**B**) type I and a (**C**) type II pyramidal cell. Scale bar 100 µm. **D**) Frequency-current curve for individual type I and type II pyramidal cells. Type I, n=46; Type II, n=30. **E**) Average frequency-current curve for type I and type II pyramidal cells. Data are presented as mean ± SEM. **E-G**) Passive characteristics of type I and type II pyramidal cells, comparing (**F**) resting membrane potential, (**G**) input resistance and (**H**) series resistance. Data are presented as boxplots with the box extending from the 25th to 75th percentiles, the middle line representing the median and the whiskers indicating the minimum and maximum values.
